# Up-regulation of circARF3 reduces blood-brain barrier damage in rat subarachnoid hemorrhage model via miR-31-5p/MyD88/NF-κB axis

**DOI:** 10.18632/aging.203468

**Published:** 2021-09-12

**Authors:** Li Cai, Beihai Ge, Shengbo Xu, Xiangwen Chen, Hong Yang

**Affiliations:** 1 Department of Neurosurgery, Guangxi International Zhuang Medicine Hospital, Nanning 530221, Guangxi, China; 2 Department of Neurology, Guangxi Zhuang Autonomous Region Brain Hospital, Liuzhou 545005, Guangxi, China

**Keywords:** circARF3, miR-31-5p, cerebral endothelial cell, subarachnoid hemorrhage, inflammation

## Abstract

Inflammation events have been found to aggravate brain injury and blood-brain barrier (BBB) damage following subarachnoid hemorrhage (SAH). This study probed the role and mechanism of a novel circRNA, circARF3, in regulating the BBB injury in SAH rats and hypoxia-induced vascular endothelial cell (VEC) injury *in vitro*. Levels of circARF3 and miR-31-5p were monitored by RT-PCR. The expression of inflammatory factors IL-1β and TNF-α was verified by ELISA. *In vivo* SAH model was constructed in Sprague Dawley (SD) rats. The BBB integrity and cerebral edema, as well as the neurological functions of the rats were evaluated. The apoptotic neurons and microglia in brain lesions were examined by immunohistochemistry (IHC). The MyD88/NF-κB pathway was tested by Western blot. Furthermore, gain-of functional assay were constructed to explore the effects of circARF3 and miR-31-5p in primary cultured brain microvascular endothelial cell (BMEC) injury and microglial inflammation induced by oxygen and glucose deprivation (OGD). circARF3 was significantly down-regulated in plasma and CSF in SAH patients with higher Fisher stages. In the SAH rat model, overexpressing circARF3 improved BBB integrity and neurological score, decreased neuronal apoptosis and microglial activation in ipsilateral basal cortex, with declined miR-31-5p expression and MyD88-NF-κB activation. *In vitro*, overexpressing circARF3 attenuated OGD-mediated integrity destruction of BMECs and microglial induced neuroinflammation, while overexpressing miR-31-5p had opposite effects. Mechanistically, circARF3 sponged miR-31-5p as an endogenous competitive RNA and dampens its expression, thus inactivating MyD88-NF-κB pathway. CircARF3 attenuates BBB destruction in SAH rats by regulating the miR-31-5p-activated MyD88-NF-κB pathway.

## INTRODUCTION

Subarachnoid hemorrhage (SAH) is a neurovascular emergency affecting relatively young adults. Cerebral trauma [1] and rupture of cerebral aneurysm [2] are the common causes of SAH. SAH accounts for only 5% of the strokes, but its mortality accounts for 25% of cerebrovascular disease deaths [3]. Many studies have shown that cerebral vasospasm is a potentially disabling or fatal complication in patients with aneurysmal SAH, which often occurs within 3-14 days after aneurysm rupture. However, the underlying mechanism of cerebral vasospasm remains unclear [4]. It’s worth noting that the blood-brain barrier (BBB) destruction leads to severe brain damage and induces brain edema in SAH patients, which is closely with brain edema after cerebral hemorrhage [5, 6]. Within 72 hours after SAH, BBB damage causes cerebral circulation insufficiency and leads to excessive oxidative stress and inflammation, further aggravating neuronal apoptosis and neurological dysfunction [7–9]. In this pathological process, microglia are selectively activated into two phenotypes, the “M1” polarized microglia release pro-inflammatory cytokines, chemokines, oxidative metabolites, etc., thus aggravating brain damage. However, the altered activated microglia devour harmful substances and improve neuron and BBB recovery [10, 11]. Hence, effective mitigation of BBB destruction and inflammation following SAH becomes a new strategy to improve the prognosis of SAH patients.

Circular RNAs (circRNAs) are a type of long non-coding endogenous RNA without protein-coding function. They are involved in a variety of genomic transcription and post-transcriptional regulation [12]. Various studies have suggested that circRNAs regulate the integrity and inflammation of vascular endothelial cells (VECs) [13, 14]. In particular, circRNAs also play a significant role in regulating stroke-induced BBB destruction and microglial inflammation. For example, circRNA DLGAP4 is significantly declined in plasma in patients with acute ischemic stroke, while its overexpression reduces neurological impairment, infarct size, and BBB damage in stroke model mice [15]. In addition, circPTK2 regulates microglia-induced neuronal apoptosis by sponging miR-29b. Also, circPTK2 contributes to regulating oxygen and glucose deprivation (OGD)-activated microglia-induced neuronal apoptosis via the circPTK2-miR-29b-SOCS-1-JAK2/STAT3-IL-1β axis [16]. As a novel circRNA, circARF3 (ADP-ribosylation factor 3) is transcribed from a gene located on chromosome 12, which was first identified by Salzman J et al. [17]. Previous studies have shown that circARF3 relieves TNF-α-mediated neurocyte cell inflammatory damage [18] and mitophagy-mediated adipose inflammation [19]. Therefore, we posited that circARF3 exerted a vital role in SAH-mediated BBB injury and microglial activation.

MicroRNAs (miRNAs) are a class of endogenous non-coding small RNAs with a length of 18~25 nucleotides. They regulate protein expression through the messenger RNA of protein-coding genes via translational inhibition or transcriptional substitution, thus playing an extensive role in cell biological functions [20]. Emerging studies have shown that miRNAs affect stroke-induced neuroinflammation [21], VEC injury [22] and neuronal apoptosis [23] by regulating microglial activation. It is noteworthy that miR-31-5p can not only directly regulate the proliferation, migration and invasion of tumor cells as a biomarker for tumor diagnosis [24], but also modulate the angiogenesis of VECs [25]. What’s more, miR-31-5p mediates endothelial dysfunction by regulating the endothelial nitric-oxide synthase, and the NF-κB activation directly leads to the up-regulation of miR-31-5p [26]. On the other hand, circRNA functions as a miRNA sponge and interacts with miR-31-5p, which targets its downstream gene and affects Renal Cell Carcinoma progression [27]. Therefore, we focused on the potential regulatory axis of circARF3/miR-31-5p in SAH.

In this study, we examined the levels of circARF3 and miR-31-5p in plasma and cerebrospinal fluid (CSF) in SAH patients. We discovered that circARF3 was lowly expressed, while miR-31-5p was overexpressed in SAH patients with higher Fisher stages, and they had a negative relationship in the plasma and CSF of SAH patients. Through bioinformatics, it was found that circARF3 has a potential targeting relationship with miR-31-5p. *In vitro*, overexpressing circARF3 significantly attenuated OGD-mediated brain microvascular endothelial cell (BMEC) integrity destruction and inflammatory response, while miR-31-5p had the opposite effects. Meanwhile, circARF3 overexpression reduced the miR-31-5p level. Therefore, we were curious whether circARF3 could play a protective role in BBB in the SAH rat model. We hope to identify a novel regulatory axis, namely the circARF3-miR-31-5p axis in SAH, so as to provide a new theoretical reference for easing the BMEC dysfunction and the imbalance of inflammation after SAH.

## MATERIALS AND METHODS

### Collection and treatment of clinical specimens

Blood and CSF of acute SAH patients (onset within 24 hours) admitted to Guangxi International Zhuang Medicine Hospital from March 2020 to June 2020 were collected within 24 hours of admission, and a total of thirty samples were obtained. The average age of thirty SAH patients was 48.51±8.25 years old, of which 16 were males and 14 were females. All participants signed written consent and agreed to get involved in this study. After sample collection, the cells and other impurities in the blood and CSF samples were removed through low-speed centrifugation (178g for 15 min at 4° C), and the collected samples were stored at -80° C. Acute SAH was diagnosed based on the patient's clinical symptoms and confirmed by CT examination, and the Fischer scoring system was used to assess the severity of vasospasm [28]. This study was approved and conducted by the Medical Ethics Committee of Guangxi International Zhuang Medicine Hospital.

### Culture of BMECs and primary microglia

Primary culture of BMECs was referred to previous study [29]. SD rats (16-day-old, 25 to 30 g) were anesthetized with phenobarbital (60 mg/kg) and then killed. The rat’s skull was cut open under aseptic conditions, and the brain was put in a culture dish containing cold phosphate-buffered saline (PBS). Afterward, the brain was dissected to remove the diencephalon, hippocampus, white matter, and gray matter, etc., and only the cerebral cortex was left. Next, the cerebral cortex was rolled on dry sterilized filter paper. After removing the arteries of pia mater and meninges, the brain was rinsed with PBS for three times and cut into 1-mm puree with a surgery scissor. Then, the purees were mixed with 0.1% (mass fraction) collagenase II and trypsinized at 37° C in the incubator for 90 min (the incubator was shaken every 10 min). Subsequently, the centrifugation (111.8 rpm, 8 min) was performed at room temperature (RT), and the supernatant was discarded. The digested product was added with 20% (mass fraction) BSA of twice the volume, gently dissociated, and centrifuged at 4° C (1000 g, 20 min). The middle and upper layers of nerve tissues and arteries were discarded, and the precipitation was retained. After that, the precipitation was supplemented with 2 mL dulbecco's modified eagle medium (DMEM) and gently dissociated for washing. Then, it was centrifuged (111.8 g, 5 min) at RT, and the supernatant was discarded. Next, 2 mL 0.1% (mass fraction) collagenase/dispase and 2 kU/mL DNase I 30 were added. The product was put into a 37° C incubator for trypsinization for 1 hour and centrifuged (55 g, 6 min) at RT, and the supernatant was discarded. The product was rewashed with 2 mL DMEM and gently dissociated five times. After centrifugation (111.8 g, 5 min) at RT, the supernatant was removed. Finally, 2 mL of 20% (mass fraction) serum albumin was added, gently dissociated ten times, and centrifuged (4° C, 1000 g, 20 min). The supernatant was discarded, and the BMECs were obtained for subsequent experiments. Cellular immunofluorescence was used for evaluating the purity of BMECs (labeled by CD31 and vWF). The first generation of BMECs had a purity about 90%. At the third generation of BMECs, the purity was over 95%.

The primary culture of microglia was according to a previous study with few changes [30]. Briefly, new-born SD rats (1–2-day, 5-6 g) were sacrificed by neck amputation, and the cerebral cortex was carefully separated. The meninges and microvascular were exfoliated using tweezers, while scissors were employed to cut the brain tissue into 1 mm^3^ pieces. Then, 0.125% trypsin was added, and the cells were dispersed with the pipette. Next, the cells were trypsinized in an incubator at 37° C for 15-20 min, and the complete culture medium was added to terminate the trypsinization. After centrifugation, the supernatant was removed, and the cells were resuspended in a fresh complete culture medium (containing 20% fetal bovine serum (FBS)) and transferred to a 100 mm culture dish for culture for 24 hours. After that, the supernatant was discarded, and the nonadherent tissues were cleaned with PBS. The complete medium containing 20% FBS was added for continuous culture for 7-10 days until the appearance of highly refracted cells on the cell surface. Subsequently, the cells were shaken at 37° C at 200 rpm for 2 hours, and the supernatant was taken for centrifugation. Finally, the cells were supplemented with the complete medium, resuspended, and transferred to the culture plate. Microglia with a purity of more than 95% were harvested.

### Cell immunofluorescence

The primary cultured and third cultured endothelial cells were respectively planted on collagen-coated glass slides. Then, they were rinsed with 0.01 mol/L PBS three times, fixed with 4% (mass fraction) paraformaldehyde at RT for 15 min, and cleared with 0.01 mol/L PBS three times. Endothelial cells were identified by immunofluorescence co-staining with anti-CD31 (ab7388, Abcam, USA) and anti-vWF (sc-365712, Santa Cruz Biotechnology, USA) antibody, the specific markers of endothelial cells. Namely, 5% (volume fraction) of goat serum was blocked at RT for half an hour, and CD31 and vWF primary antibody mixtures were added and incubated at 4° C overnight. The serum was cleaned with PBS three times, and Alexa Fluor 594 and Alexa Fluor 488 secondary antibody mixtures were added for incubation at RT for 1 hour. Afterward, the serum was rewashed (three times). The slides were mounted with DAPI mounting medium and then observed under a fluorescence microscope (Olympus, Japan).

### Cell transfection

BMECs and Primary microglia in good growth condition were seeded in 6-well plates (5×10^6^/well), and the transfection was conducted after stable cell growth. circARF3 overexpression plasmids, miR-31-5p mimics and the corresponding negative controls were constructed and synthetized by GenePharma (Shanghai, China). Those expression vectors were transfected into primary cultured microglia and BMECs using Lipofectamine®3000 (Invitrogen; ThermoFisherScientific, Inc., USA) according the instructions of the manufacturer. The cells were incubated at 37° C with 5% CO_2_. Twenty-four hours after the transfection, the total RNA was isolated for reverse transcription-polymerase chain reaction (RT-PCR) to confirm the transfection efficiency.

### Establishment of the oxygen-glucose deprivation/reoxygenation (OGD/R) model *in vitro*

The OGD/R model was used to simulate the *in vitro* damage model of BMECs and the inflammatory activation model of microglia. Simply, BMECs were seeded in 6-well plates at 5×10^6^/well. Glucose-free DMEM was pre-balanced at 37° C in an incubator with 1% O_2_, 5% CO_2_ and 94% N_2_. The complete primary medium was discarded, and BMECs were washed with glucose-free DMEM three times. Subsequently, BMECs were cultured in the glucose-free DMEM medium and transferred to an incubator containing 1% O_2_, 5% CO_2_ and 94% N_2_ for 1.5 hour at 37° C. The medium was then replaced with a fresh, complete medium and cultured in a normal incubator for 4 hours to detect cell viability and integrity.

### Dual-luciferase reporter assay

Online database Starbase was adopted to predict the binding sites of circAFR3 and miR-31-5p. The 3 'untranslated region of circARF3 was amplified from the total DNA of the human genome and cloned into the downstream of the reporter vector pMIR-circAFR3-WT luciferase reporter gene. Meanwhile, the pMIR-circARF3-MUT vector was constructed, which mutated the binding site of miR-31-5p and circARF3. All luciferase reporter vectors were constructed and synthesized by Promega Company (Madison, WI, USA). 293T cells were inoculated in 6-well plates with 5×10^6^ cells/well. The next day after transfection, pMIR-circAFR3-WT, pMIR-circARF3-MUT and miR-31-5p mimics or miR-NC were transfected. Forty-eight hours later, the luciferase activity was verified by a dual-luciferase system (Promega).

### Cell viability test

The CCK-8 assay was implemented to verify cell viability. The stably transfected cells were seeded into 96-well plates (1×10^3^ cells/well) and incubated for 24 hours. Then, 10 μL CCK8 (Dojindo Molecular Technologies, Kumamoto, Japan) reagent was added to each well and incubated at 37° C for 1 hour as per the manufacturer's guidelines. The OD value at 450 nm was measured on a spectrophotometer (Bio-RAD, CA, USA).

### RT-PCR

The circARF3 and miR-31-5p profiles were tested. The TRIzol reagent (Invitrogen, Carlsbad, CA, USA) was adopted to routinely extract total cellular RNA, and the genomic DNA was removed using Deoxyribonuclease I. RT-PCR was carried out with the reverse transcription kit. The conditions were as follows: 10 min at 70° C, 5 min on ice, 60 min at 42° C, 5 min at 95° C, and 5 min at 0° C. The RT-PCR system was 25 μL, containing 500 ng cDNA template, 250 nmol/L forward and reverse primers, and 12.5 μL 2×SYBR Green PCR Master mixture. The reaction tube was placed into the MX3000P RT-PCR instrument. The reaction conditions were as follows: 40 s at 94° C, 40 s at 55° C, and 40 s at 72° C, with 45 cycles, and the fluorescence signal was monitored. The 2-ΔΔCt value represented the relative gene expression, and the ct value represented the amplification cycles when the fluorescent signal of the amplified product reached the set threshold. ΔΔCt = sample to be tested (Ct target gene-Ct GAPDH)-control group (Ct target gene-Ct GADPH).

### Western blot (WB)

100 mg ipsilateral basal cortex in the rats of different groups were collected. 400 μL RIPA lysis was added for the lysing of proteins on ice. 30 min later, the lysates were collected and subjected to centrifugation (4° C, 12000r/min) for 15min. The supernatant of the protein was collected. For the protein isolation of primary BMECs and microglia, the cells were washed with PBS (0.01M, pH 7.2-7.3) 3 times for removing culture, which were then lysed with 100~200 μL RIPA lysis on ice for 30 min. Then the lysates were collected and subjected to centrifugation (4° C, 12000r/min) for 15min, The supernatant of the protein was collected. The protein concentration was determined by the BCA kit (Beyotime, Shanghai, China). Proteins were separated with SDS-PAGE, and 50 μg of which were loaded to each well. After 2 hours of electrophoresis, the proteins were transferred to PVDF membranes by the wet method. Afterward, the membranes were blocked with 5% skimmed milk for 1 hour and incubated at 4° C. They were then incubated at 4° C overnight with the primary antibodies, including myeloid differentiation factor 88 (MyD88) antibody (ab133739) (1:1000, Abcam), nuclear factor kappa-B (NF-κB) antibody (ab32360) (1:1000, Abcam), NF-κB p65 (phospho S536) antibody (ab239882) (1:1000, Abcam), ZO1 antibody (ab221546) (1:1000, Abcam), Occludin antibody (ab167161) (1:1000, Abcam), and Claudin 5 antibody (ab131259) (1:1000, Abcam). The next morning, TBST was utilized to rinse the membranes, which were incubated with horseradish peroxidase-labeled secondary antibody (ab205718, 1:2000, Abcam) at 37° C for 1 hour. The membranes were developed with IMMOBILON WESTERN CHEMILUM HRP SUBSTRATE (WBKLS0100, Millipore) and the image was saved. Using Quantity One (V4.6.6) to determine the optical density value. The experiment was repeated three times.

### Enzyme-linked immunosorbent assay (ELISA)

Human plasma, CSF and rat brain tissue homogenate were centrifuged at a radius of 13.5 cm and at 3000 rpm for 15 min. The contents of IL-1β and TNF-α in the supernatant were measured by ELISA using commercially purchased ELISA kits (70-EK206/3-96, 70-EK106/2-96, 70-EK282/3-96, 70-EK182HS-96) (MultiSciences, Hangzhou, China). All procedures were performed according to the producer’s instructions. and the results were expressed in terms of the number of picograms of cytokines per gram of protein, namely pg/g (total protein).

### BMEC permeability experiment

1 mg FITC-dextran 20 was dissolved in DMEM medium containing 0.1% BSA to make a 0.01% working solution. The stably transfected BMECs were inoculated into a Transwell chamber (4 μM pore diameter) (1×10^4^ cells/well) (Corting, NY, USA). After 72 hours, the medium was removed, and the Transwell upper chamber liquid was replaced by 0.01% FITC-dextran 20. After 0 min, 30 min, 60 min, and 120 min, 50 μL of the Transwell lower chamber liquid was aspired into an EP tube and stored at -80° C. The fluorescence intensity of each group was determined with a microplate reader.

### Establishment of the rat SAH model

A total of 80 SD rats (8 weeks old, 220 to 250 g) were provided from the animal center of Guangxi Medical University. The rats were anesthetized through intraperitoneal injection of phenobarbital (60 mg/kg), and then they were in the supine position, with their limbs fixed for preoperative preparation. The left median cervical incision was performed to fully expose and separate the left common carotid artery, internal carotid artery and external carotid artery. The distal end of the external carotid artery was ligated, and the common carotid artery and internal carotid artery were temporarily blocked with a carotid clamp. A small incision was made at the proximal end of the external carotid artery ligation, and a nylon thread with a diameter of 0.2 mm was inserted. The external carotid artery was cut and pulled to form a straight line with the internal carotid artery, and the nylon thread was inserted into the internal carotid artery to the bifurcation. After encountering resistance, inserting the nylon wire with a little force until reaching a breakthrough. Then, the nylon wire was pulled out, the proximal end of the external carotid artery was ligated (in the sham group, no nylon wire was inserted), the artery clamp was loosed, and the incision was sutured. The animal procedures were shown in Supplementary Figure 1. 30 min after the successful construction of rat SAH model, the rats were fixed on a stereotactic instrument (AP: -0.6mm, ML: ± 1.3mm, DV: ± 1.2mm). Craniotomy was performed, and mucous membranes were removed with hydrogen peroxide to expose the anterior fornix. After positioning, the skull was drilled. The skull was then blocked, and the wound was sutured. Overexpressed lentivirus of CircARF3 or the negative control (constructed and synthesized by Invitrogen, Carlsbad, CA, USA) were injected with a microsyringe at 1min/μl for 5 min. Finally, the rats were caged and fed. The Hematoxylin and Eosin Staining kit (Beyotime, Shanghai, China) was used for evaluating the pathological changes of the brain lesions.

### Water maze experiment

10 rats in each experimental group were randomly selected for conducting Morris water maze experiment to observe the behavioral changes of animals, specifically referring to the method of Yao et al. Briefly, the round stainless-steel pool (100 cm in diameter and 50 cm in height) was divided into four quadrants, and a circular hidden platform with a diameter of 9 cm and a height of 27 cm was placed in the center of the target quadrant. The water temperature in the pool was kept at (22±1)° C, and the water was 1 cm above the platform. A proper amount of milk powder was added to the water to hide the platform. The motion track of mice was recorded by a camera above the pool and analyzed by the Ethovision XT monitoring and analysis system. The experiment included a place navigation test and a spatial probe test.

### Modified garcia score

Neurological function was tested 24 hours, 3 days, 7 days and 14 days after the modeling according to the modified Garcia score [31]. The neurological score was conducted from six aspects, including spontaneous activity, limb motion symmetry, forefoot extension, climbing, contact reaction and tentacle reaction to the bilateral trunk. The lowest score for each was 0, and the highest score was 3, with a full score of 18. The scoring was done independently by two uninformed persons, and the average value was taken.

### Determination of cerebral edema

The water content of the brain tissue was calculated by the dry/wet method. About 100 mg of the brain tissue was taken, weighed on an electronic balance, and baked in an oven at 105° C for 48 hours to constant weight. Water content of the brain tissue (%) = (wet weight - dry weight)/wet weight ×100%, the accuracy was 0.2 mg.

### Evans blue staining

Referring to previous research methods [32], we evaluated the cerebrovascular integrity of C57BL/6J mice. 2% Evans blue solution (4 mL/kg) was injected into the lateral caudal vein to assess cerebrovascular permeability. After four hours, the mice were anesthetized and perfused in the left ventricle with cold PBS (pH 7.4) to remove Evans blue in blood vessels. The brain was collected, sectioned, and scanned before homogenization in PBS (1:10g/v). The homogenate was precipitated in 15% trichloroacetic acid (1:1 v/v) and centrifuged (1000 rpm, 10 min). PH value was adjusted by adding 125 μL sodium hydroxide to the 500 μL supernatant. The concentration of Evans blue was measured by spectrophotometry at 620 nm.

### TdT-mediated dUTP nick end labeling (TUNEL) experiment

Neuronal apoptosis in the ipsilateral basal cortex was detected by the TUNEL kit (Beyotime Biotechnology Co., Ltd., Wuhan, China). 24 hours after SAH, the rats were sacrificed and the brains were isolated and fixed with 4% neutral formaldehyde for 24 hours (n=5 in each group). Then the brains were embedded in paraffin. After that, the brains were sectioned (4 μm) and the paraffin brain sections were dewaxed in xylene for 5-10 minutes and then fresh xylene was provided for an equal time of dewaxing. The sections were then soaked in absolute ethyl alcohol for 5 minutes, 90% ethanol for 2 minutes, 70% ethanol for 2 minutes, and distilled water for 2 minutes. Subsequently, 20 μg/mL DNase-free protease K (Beyotime Biotechnology Co., Ltd., Wuhan, China) was added and maintained at 20-37° C for 15-20 minutes. 20 μg/mL DNase-free protease K was obtained by 1000 times dilution of P0106 immunostaining wash solution or 10 mM Tris-HCL (pH 7.4-7.8). Next, the sections were washed with PBS or HBSS three times. The appropriate amount of TUNEL test solution was prepared according to the instructions, which was well mixed. Note: the prepared TUNEL test solution must be used all at once and cannot be frozen. Finally, the TUNEL-positive cells in the ipsilateral basal cortex were observed using a microscope (Olympus cx31, Tokyo, Japan).

### Immunohistochemistry (IHC)

IHC was conducted to verify Iba1 (a microglial marker in the brain) expression in the ipsilateral basal cortex. Paraffin-embedded sections were dewaxed with xylene and hydrated with gradient ethanol. Then, paraffin sections were immersed in 10 mm citrate buffer (pH6.0) and boiled in a pressure cooker at 121° C for 4 minutes to recover the antigen. The sections were then cooled slowly at RT, soaked in a 10 mm citric acid buffer, and rinsed with an appropriate amount of PBS. The sections were treated with 3% H_2_O_2_ for 15 minutes at RT to block the endogenous peroxidase activity and then incubated with the anti-Iba1 (1:200; ab178846; Abcam) at 4° C overnight. After that, the Goat Anti-Rabbit IgG H&L (Horseradish Peroxidase, HRP) (1:500, ab205718; Abcam) was added and incubated at RT for 0.5 hour. The immunohistochemical staining was performed with 3, 5-diaminobenzidine (DAB, Maxim), and the sections were observed and photographed under a light microscope. Image Pro 6.0 software (Media Controlnetics, Inc., Rockville, MD, USA) was used for quantitative analysis of immunohistochemical staining.

### Statistical analysis

SPSS17.0 statistical software (SPSS Inc., Chicago, IL, USA) was employed for analysis. Measurement data were expressed as mean ± standard deviation (x±s). The correlation test was analyzed by the Pearson correlation test. Univariate analysis of variance was adopted for multi-factor comparison, and *t* test was used for pair-wise comparison. *P*<0.05 indicated statistical significance.

### Ethics statement

Our study was approved by the Medical Ethics Committee of Guangxi International Zhuang Medicine Hospital.

### Data availability statement

The data sets used and analyzed during the current study are available from the corresponding author on reasonable request.

## RESULTS

### CircARF3 was down-regulated in plasma and CSF of SAH patients

**Figure 1:**
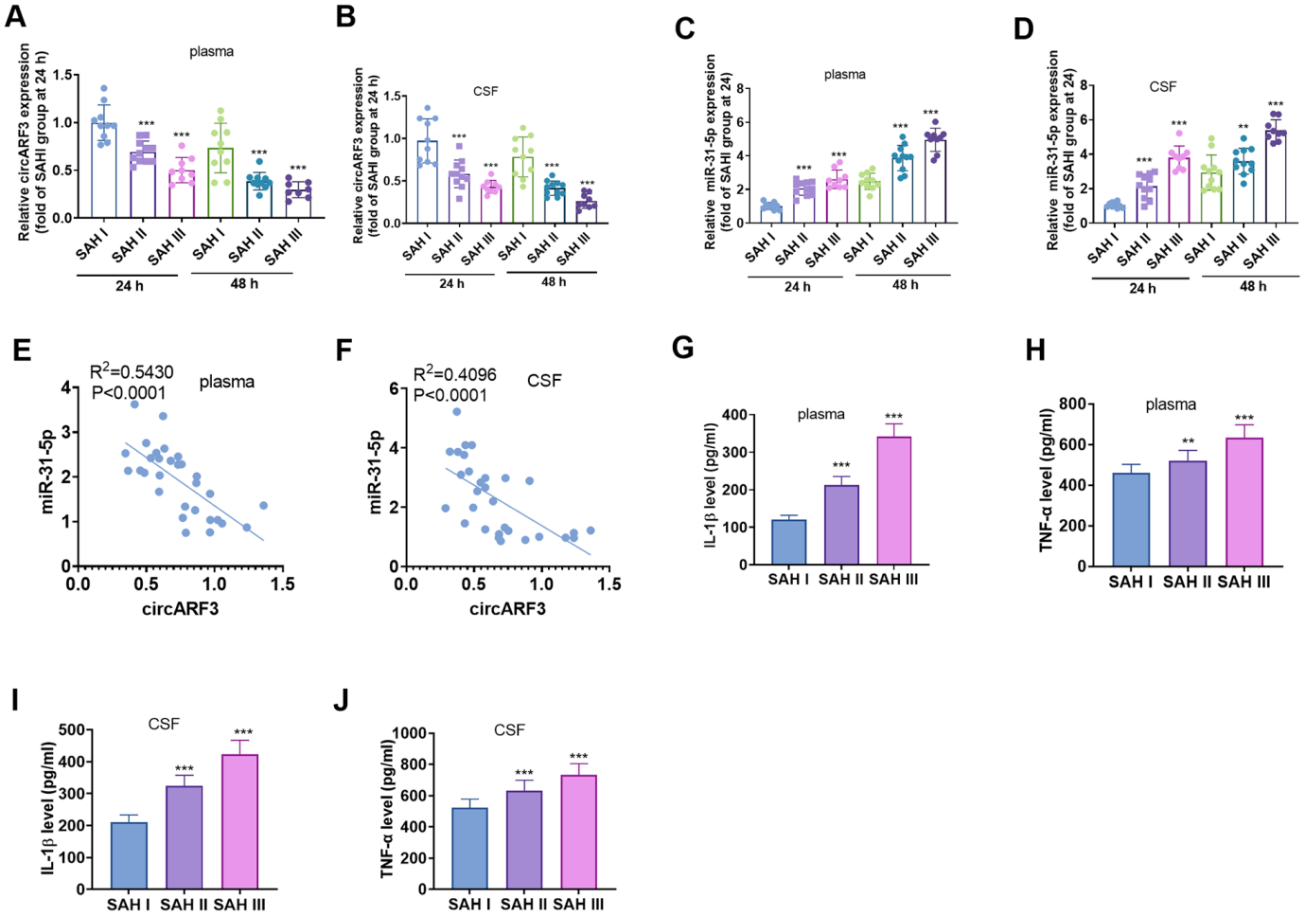
**CircARF3 was down-regulated in plasma and CSF of SAH patients.** (**A**–**D**) RT-PCR was used to detect the changes of circARF3 (**A**, **B**) and miR-31-5p (**C**, **D**) in plasma and cerebrospinal fluid (CSF) of patients with different grades of SAH. (**E**, **F**) The correlation between circARF3 and miR-31-5p in plasma and CSF of SAH patients was analyzed by Pearson linear regression. (**G**–**J**) Levels of IL-1β and TNF-α in plasma and CSF samples of SAH patients were determined by ELISA. * * * indicated that *P* < 0.001. Each experiment was repeated for 3 times.

RT-PCR was performed to evaluate circARF3 and miR-31-5p alterations in the serum and CSF of SAH patients. The results revealed that circARF3 expression decreased significantly in plasma in SAH patients at Fisher stage II and III compared with that in SAH patients at Fisher stage I (Figure 1A, 1B). Meanwhile, miR-31-5p levels were significantly increased in plasma of SAH patients at Fisher stage II and III compared with that in SAH patients at Fisher stage I (Figure 1C, 1D). Among the SAH patients, the Pearson linear regression analysis showed that circARF3 was reversely correlated with miR-31-5p in plasma and CSF of SAH patients (Figure 1E, 1F). Further, the levels of IL-1β and TNF-α in plasma and CSF samples were analyzed by ELISA. As a result, their levels were significantly higher in the plasma of SAH patients with higher Fisher stages (Figure 1G–1J), and their level reached the highest level in the SAH patients at Fisher III stage (Figure 1G–1J). These results suggested that both circARF3 and miR-31-5p were involved in SAH-induced inflammation.

### Overexpressing circARF3 attenuated nerve function injury and neuronal apoptosis in SAH rats

**Figure 2:**
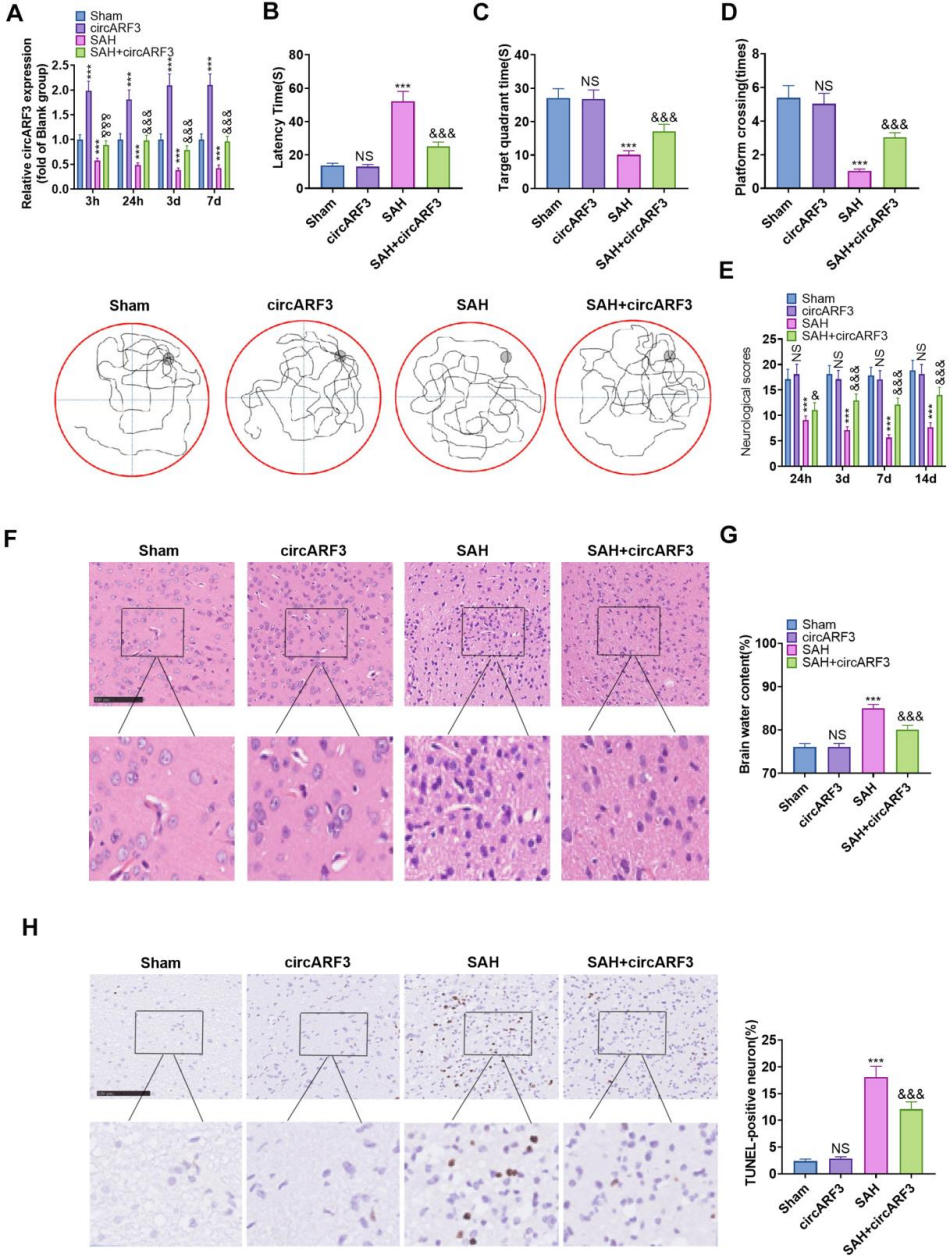
**Overexpressing circARF3 attenuated nerve function injury and neuronal apoptosis in SAH rats.** The circARF3 overexpression rat model was constructed using lentivirus-carried circARF3 vectors, and the SAH rat model was also constructed. (**A**) The circARF3 expression in rat brain tissues at different time points (3 hours, 24 hours, 3 days, and 7 days) was verified by RT-PCR. (**B**–**D**) On the 16th day of SAH, the water maze experiment was conducted to evaluate the learning and memory function of rats. (**E**) The modified Garcia score was applied to assess the motor function in rats. (**F**, **G**) Changes of cerebral edema were determined by HE staining (**F**) and wet/dry method (**G**). (**H**) TUNEL assay was implemented to detect the apoptosis rate of neurons in brain lesions. NS, ***P>0.05, *P*<0.001 vs. Sham group. &, &&&*P*<0.05, *P*<0.001 vs. SAH group. N=10. Scale bar=100 μm.

We used lentivirus-carried circARF3 vectors to construct a circARF3 overexpression rat model and established a SAH rat model. RT-PCR was conducted to test the circARF3 expression in the brain tissue. It turned out that the circARF3 level in the circARF3 group was significantly heightened (vs. the sham group). In SAH rats, the level of circARF3 was significantly lower than that of the blank group (Figure 2A). Next, we evaluated learning, memory, and motor functions in rats using the water maze experiment and modified Garcia scores. The results demonstrated that the SAH group's latency of finding the platform, the number of times of crossing the platform, and the residence time in the platform were significantly shortened (Figure 2B–2D), while the modified Garcia score was reduced (Figure 2E). However, after circARF3 overexpression, the rats' performance in the water maze experiment was improved, and the modified Garcia score was elevated (vs. the SAH group, Figure 2B–2E). Then, we measured the changes of cerebral edema by Hematoxylin and Eosin (HE) Staining and dry/wet method. It turned out that the brain edema increased significantly in SAH rats, while it was alleviated by overexpression of circARF3 (Figure 2F, 2G). The apoptosis rate of neurons in the brain injury lesions was determined by TUNEL assay. It was found that the number of TUNEL positive cells in SAH model rats was elevated, while it was declined after circARF3 overexpression (Figure 2H). Based on the above results, we concluded that circARF3 overexpression had a neuroprotective effect in the SAH rat model.

### Overexpressing circARF3 eased SAH-induced BBB injury and MyD88-NF-κB pathway-mediated inflammation

**Figure 3:**
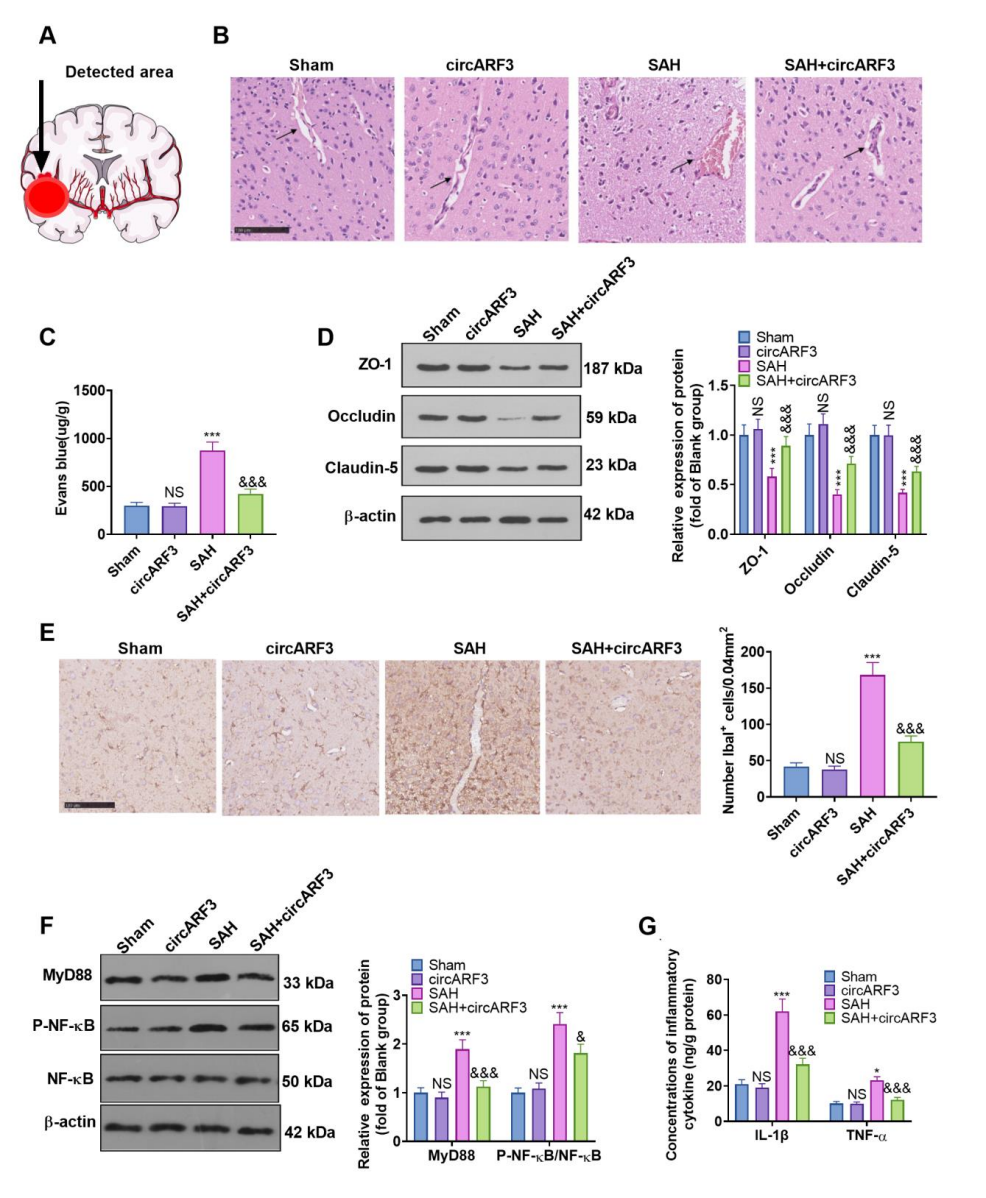
**Overexpressing circARF3 alleviated SAH-induced BBB injury and MyD88-NF-κB pathway-mediated inflammation.** (**A**) The ipsilateral basal cortex was used for histopathological examination. (**B**, **C**) The integrity of BBB was detected by HE staining and Evans blue staining. (**D**) The expression of ZO-1, Occludin, and Claudin-5 in BMECs was examined by WB. (**E**) IHC was applied to verify microglial/macrophages activation (labeled by Iba1). (**F**) The MyD88-NF-êB pathway activation was examined by WB. (**G**) The expression of IL-1â and TNF-á in the brain lesions was monitored by ELISA. NS, *, ***P>0. 05, P<0.05, P<0.001 vs. Sham group. &, &&&P<0.05, P<0.001 vs. SAH group. N=6.

We tested the integrity of BBB with HE staining and Evans’ blue to further explore the protective mechanism of circARF3 in attenuating brain injury in SAH rats. The detected area was shown in Figure 3A. The results confirmed that after SAH, the integrity of BMECs in injury lesions was destroyed, and a large number of blood cells exuded from brain tissues (Figure 3B). Meanwhile, the permeability of Evans blue in brain tissue was significantly increased (Figure 3C). Next, the expression of tight junction proteins (ZO-1, Occludin, and Claudin-5) in endothelial cells was compared by WB. The results confirmed that their levels in the SAH group were lower than those of the rats in sham group and were dramatically facilitated after overexpressing circARF3 (vs. the SAH group, Figure 3D). We then assessed the microglial/macrophage activation and inflammation in brain injury lesions. It turned out that SAH induced microglial/macrophage activation (Figure 3E), accompanied by the activation of MyD88-NF-κB (Figure 3F) and up-regulation of IL-1β and TNF-α (Figure 3G). In contrast, after circARF3 overexpression, the above reactions were significantly weakened (Figure 3E–3G). Thus, circARF3 exerted a neuroprotective role by attenuating BBB destruction and microglia-mediated inflammation.

### CircARF3 competitively sponged miR-31-5p

**Figure 4:**
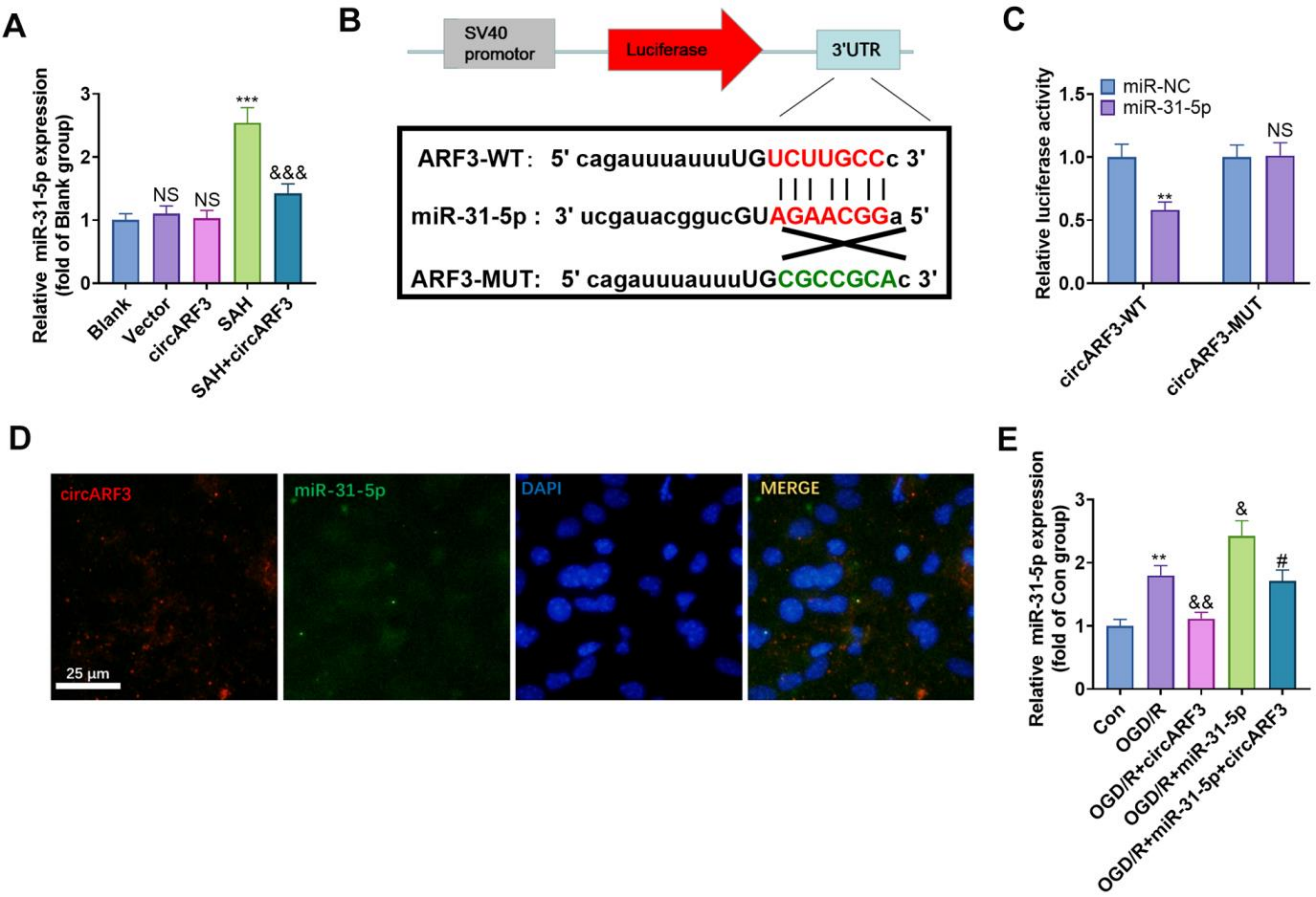
**CircARF3 competitively sponged miR-31-5p.** (**A**) The miR-31-5p expression in rat brain tissues was verified by RT-PCR, NS *P*>0.05 vs. Blank group, *** *P*<0.001 vs. Vector group. &&& *P*<0.001 vs. SAH group. (**B**) Through the Starbase, we also discovered that miR-31-5p bound to circARF3, and the binding sites between them were shown. (**C**) Dual-luciferase reporter gene assay was adopted to determine the binding relationship between miR-31-5p and circARF3, NS*P*>0.05, ** *P*<0.001 vs. miR-NC group. (**D**) The FISH experiment revealed that circARF3 bound to miR-31-5p in the cytoplasm. (**E**) CircARF3 overexpression plasmids and miR-31-5p mimics were transfected into BMECs, respectively, and the expression of miR-31-5p in BMECs was determined by RT-PCR. ** *P*<0.01 vs. Con group, & *P*<0.05, && *P*<0.01 vs. OGD/R group; #*P*<0.05 vs.OGD/R+miR-31-5p. N=3.

Previous human experiments suggested a regulatory relationship between miR-31-5p and circARF3. We implemented RT-PCR and found that the level of miR-31-5p was significantly elevated in the rat brain tissue after SAH, while it was decreased after circARF3 overexpression (vs. the SAH group, Figure 4A). Through the Starbase, we also discovered that miR-31-5p bound to circARF3 in chr12 (49330665-49330685[-]) (Figure 4B). Besides, by performing the dual-luciferase reporter assay, we discovered that the miR-31-5p mimic significantly reduced luciferase activity of 293T cells transfected with circARF3-WT, while it had no significant effect on that of cells transfected with the circARF3-MUT vector (Figure 4C). Then, the fluorescence *in situ* hybridization (FISH) experiment revealed that circARF3 bound to miR-31-5p directly in the cytoplasm (Figure 4D). Moreover, we transfected the circARF3 overexpression plasmids and miR-31-5p mimics into BMECs, respectively. Interestingly, after overexpressing circARF3, miR-31-5p level was significantly decreased, and circARF3 directly hampered miR-31-5p levels in cells transfected with miR-31-5p mimics (Figure 4E). The above results confirmed that circARF3 competitively sponged miR-31-5p and directly abated its expression.

### CircARF3 attenuated OGD/R-mediated integrity destruction of BMECs by sponging miR-31-5p

**Figure 5:**
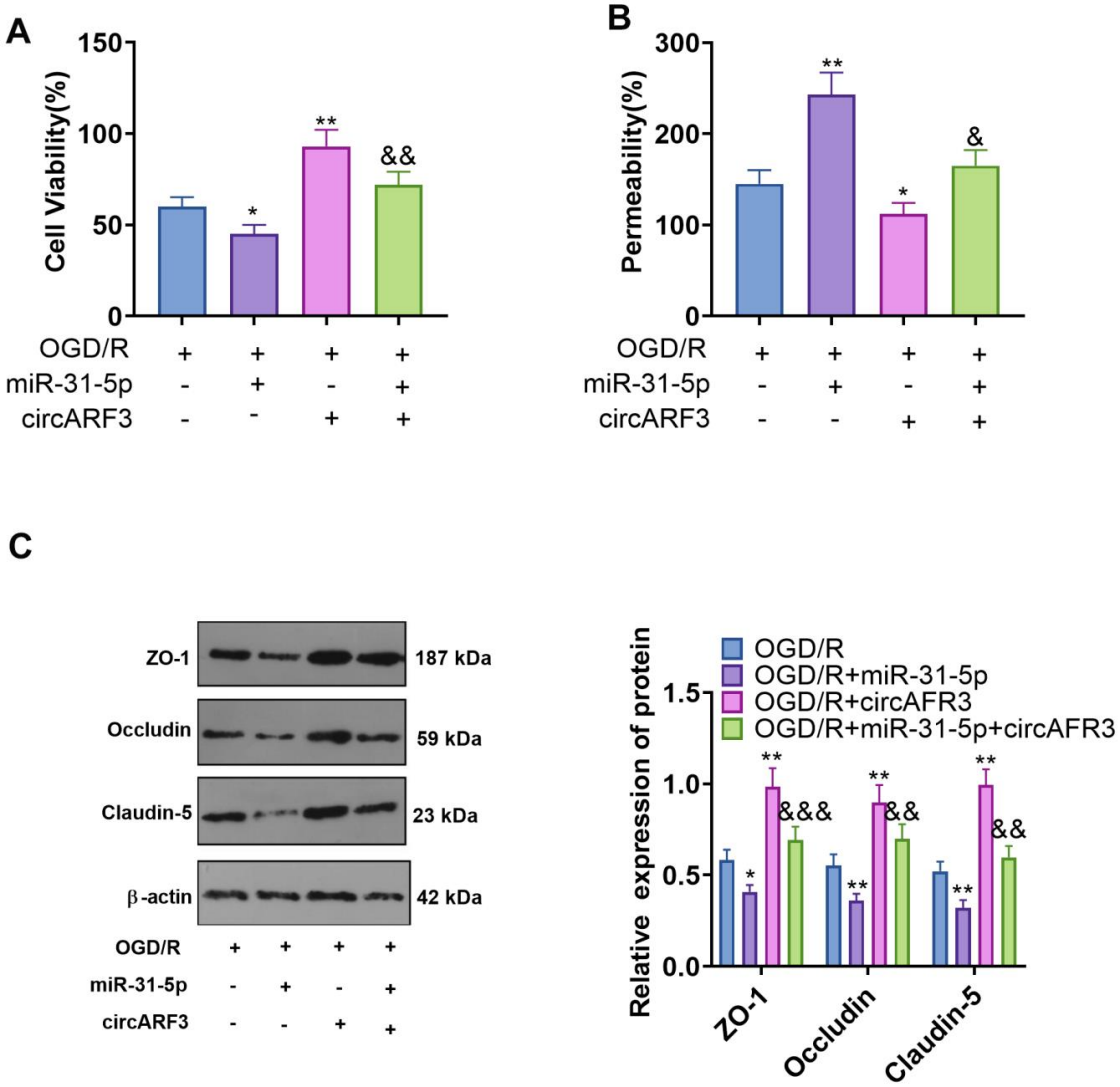
**CircARF3 attenuated OGD/R-mediated integrity destruction of BMECs by sponging miR-31-5p.** Primary BMECs were transfected with miR-31-5p mimics and/or circARF3 overexpression plasmids and then treated with OGD/R for 4 hours. (**A**) The activity of BMEC was detected by the CCK8 experiment. (**B**) The integrity of BMECs was evaluated by the permeability test. (**C**) The levels of ZO-1, Occludin and Claudin-5 were compared by WB. *, **P<0.05, *P*<0.01 vs. OGD/R group. &, &&, &&&P<0.05, *P*<0.01, *P*<0.001 vs. OGD/R+miR-31-5p group. N=3.

BMECs were isolated from new-born SD rats and identified by cellular immunofluorescence (labeled by vWF) (Supplementary Figure 2A). Then BMECs were transfected with miR-31-5p mimics and/or circARF3 overexpression plasmids were treated with OGD/R. First, we tested the viability of BMECs by the CCK8 experiment. The results confirmed that compared with the OGD/R group, cell viability decreased in the OGD/R+miR-31-5p group and increased in the OGD/R+circAFR3 group. In contrast, the cell viability of the OGD/R+miR-31-5p+circAFR3 group was higher than that of the OGD-R+miR-31-5p group (Figure 5A). Next, we employed the BMEC permeability experiment and found that miR-31-5p significantly strengthened the permeability of BMECs, while overexpressing circARF3 dampened the OGD/R-mediated increase of BMEC permeability and greatly attenuated the miR-31-5p-mediated effect (Figure 5B). In addition, the levels of ZO-1, Occludin and Claudin-5 were examined by WB. As a result, their expression was hampered by miR-31-5p while it was enhanced by circARF3 (Figure 5C). Meanwhile, circARF3 reversed the effect of miR-31-5p (Figure 5C). Thus, the up-regulation of miR-31-5p aggravated the OGD/R-mediated BMEC injury, and circARF3 could largely weaken the miR-31-5p-mediated effect.

### The regulation of CircARF3/miR-31-5p on OGD/R-mediated microglial inflammation

**Figure 6:**
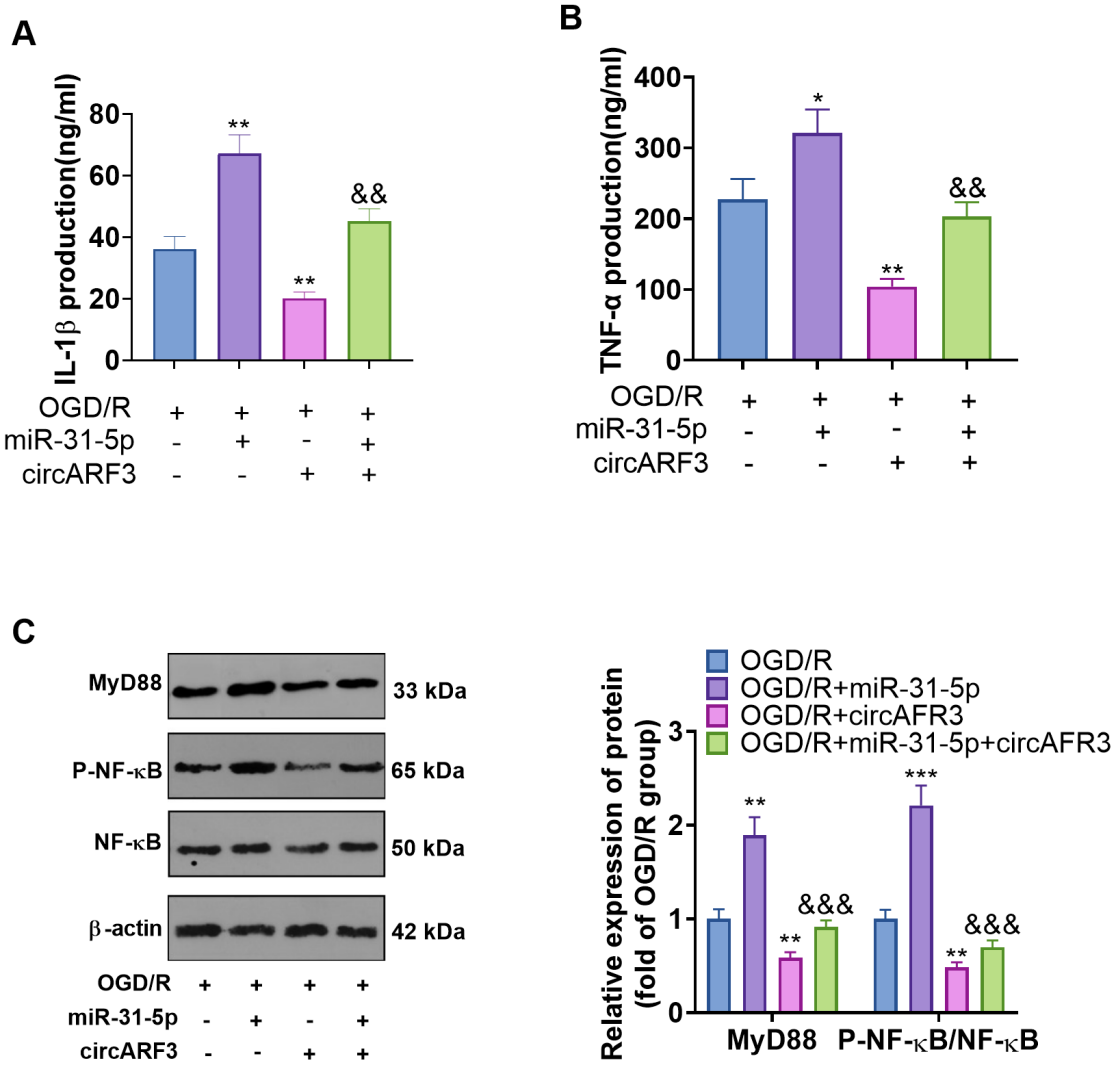
**The role of circARF3/miR-31-5p axis on OGD/R-mediated microglial inflammation.** (**A**, **B**) Primary microglia transfected with miR-31-5p mimics and/or circARF3 overexpression plasmids were treated with OGD/R for 4 hours. The levels of IL-1â and TNF-á in the medium supernatant were determined by ELISA. (**C**) The MyD88-NF-êB pathway activation in microglia was tested by WB. *, **P<0.05, P<0.01vs. OGD/R group. &&, &&& P<0.01, P<0. 001 vs. OGD/R+miR-31-5p group. N=3.

**Figure 7:**
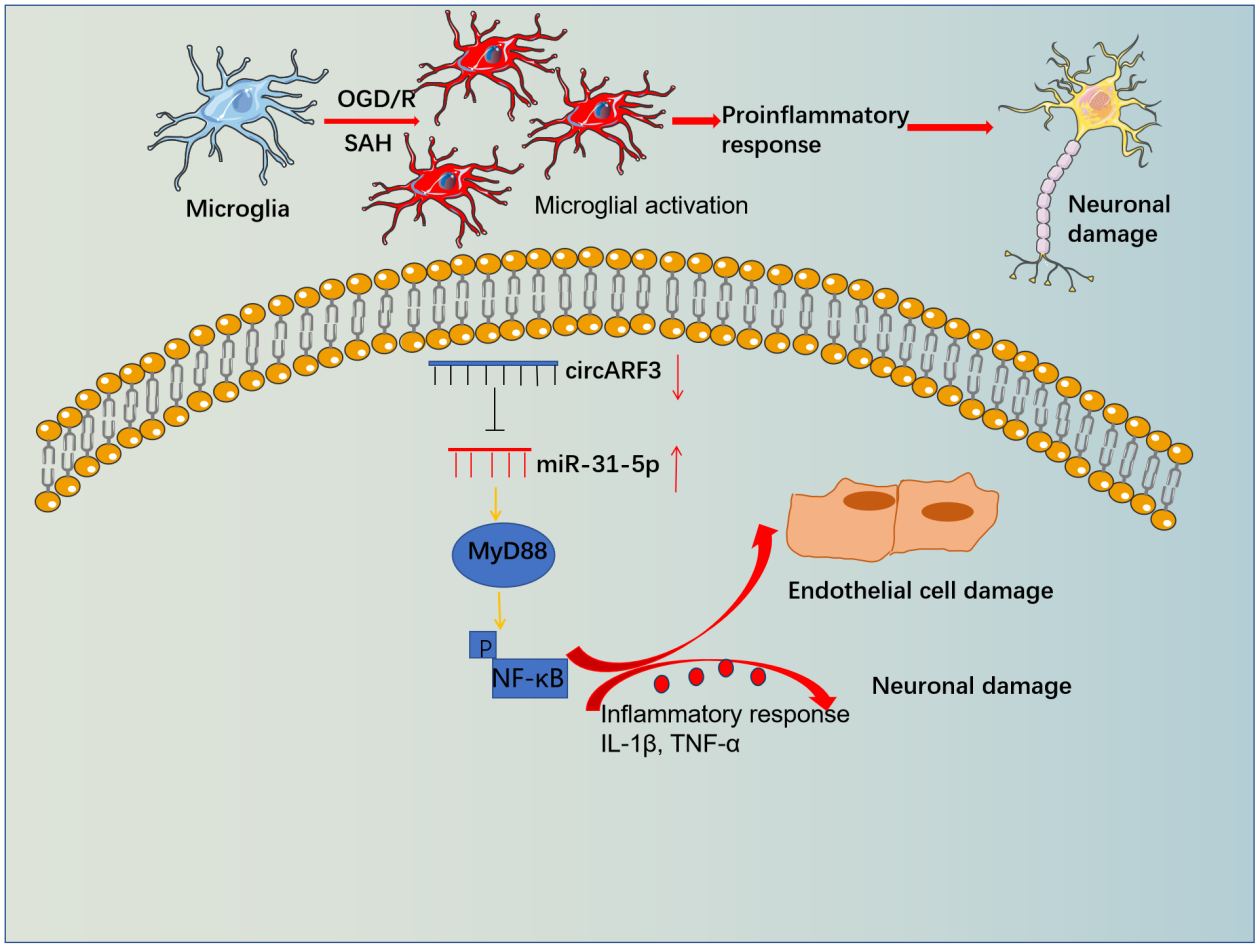
**The mechanistic diagram.** Microglia become activated and released overproduced inflammatory mediators, which then induced BBB damage and neuronal apoptosis. Forced overexpression of circARF3 attenuates BBB destruction, neuronal apoptosis in SAH rats by regulating the miR-31-5p-activated MyD88-NF-κB pathway in microglia.

Primary microglia were isolated from new-born SD rats and identified by cellular immunofluorescence (labeled by CD11b) (Supplementary Figure 2B). We determined the levels of inflammatory cytokines IL-1β and TNF-α in the medium supernatant to further probe the regulation of circARF3/miR-31-5p on microglial inflammation. As a result, compared with the OGD-R group, the levels of IL-1β and TNF-α in the OGD-R+miR-31-5p group were significantly boosted, while their levels were notably diminished in the OGD-R+circAFR3 group (Figure 6A, 6B). Besides, circAFR3 attenuated miR-31-5p up-regulation-mediated increase in IL-1β and TNF-α (Figure 6A, 6B). Moreover, the MyD88-NF-κB pathway activation in microglia was determined by WB. It turned out that compared with the OGD/R group, the phosphorylation levels of MyD88 and NF-κB were significantly heightened in the OGD-R+miR-31-5p group and dampened in the OGD-R+circAFR3 group (Figure 6C). Interestingly, their phosphorylation levels were lower in the OGD/R+ miR-31-5p +circAFR3 group than that in the OGD/R+miR-31-5p group (Figure 6C). These results illustrated that circARF3 weakened the miR-31-5p-mediated pro-inflammatory effects via repressing MyD88/NF-κB pathway (Figure 7).

## DISCUSSION

SAH is a common type of stroke and is among the leading causes of death worldwide, and dysregulated neuroinflammation has been found to exert a prominent role in brain injury following SAH [33–35]. This study investigated the regulatory effects of circARF3/miR-31-5p on SAH-induced BBB destruction both *in vitro* and *in vivo*. We found that overexpressing circARF3 inhibited miR-31-5p and improved the neurological behaviors of SAH rats via reducing BBB damage and microglial activation.

BBB, composed of endothelial cells, pericytes, astrocytes, basement membranes and extracellular matrix, can protect the brain from the damage caused by pathogens, toxins and other harmful substances in the blood [36, 37]. Functionally, BBB protects the balance within the central nervous system, and BBB disruption is a sign of severe brain diseases, including SAH [38]. During the progression of SAH, the brain edema is one of the main factors threatening the life of patients, and vasogenic brain edema caused by BBB damage is the main type of brain edema after cerebral hemorrhage [39]. BBB disruption correlates with delayed cerebral ischaemia, and the BBB permeability imaging is a potential non-invasive prognostication tool for predicting the outcomes following aneurysmal subarachnoid hemorrhage (aSAH) [40]. It has been found that mitigating BBB injury following SAH makes a role in treating SAH. For instance, the upregulation of angiogenic factor with G-patch and FHA domain 1 (Aggf1) has anti-inflammatory effect and protective effect against vascular integrity damage via the PI3K/Akt/NF-κB pathway [41]. Here, we also observed obvious BBB destruction in SAH rats’ brain tissues, with significantly increased brain edema and neuronal apoptosis. Thus, we further evaluated whether circARF2-miR-31-5p axis makes a role in BBB protection after SAH.

Emerging evidence has shown that there are a large number of circRNAs changes in cerebral injury lesions after stroke. These abnormally expressed circRNAs can not only serve as biomarkers of disease progression but also directly modulate BBB integrity after stroke [15, 42]. The blood brain barrier is assembled by the ultrastructure of BMEC, and the cells connect with each other through the junction complex of tight junction and adhesion junction [43]. Liu w et al. showed that in normal and oxygen glucose derived / recovered primary brain microvascular endothelial cells, 211 of the circRNAs were up-regulated and 326 were down regulated [44]. In addition, circRNAs have been shown to regulate a variety of biological functions of vascular endothelial cells, including proliferation, apoptosis, migration, angiogenesis and so on. For example, circdlgap4 can regulate the expression of hectd1 through miR-143, thereby reducing endothelial dysfunction induced by ischemia/reperfusion (I/R) injury [45]. In the present study, we constructed a circARF3 overexpression rat model and found that the BBB permeability of SAH rats was significantly improved after circARF3 overexpression. Meanwhile, circARF3 overexpression also eased neurological impairment, neuronal apoptosis and brain edema. These results confirmed the neuroprotective effect of circARF3.

After SAH, significant neuroinflammatory responses often emerge around the brain lesions [7–9]. The inflammatory process after intracranial hemorrhage involves infiltration of granulocytes and macrophages, activation of microglia and astrocytes, and the resulting inflammatory mediators, including cytokines, reactive oxygen species and MMP [46]. Within 4 hours after intracranial hemorrhage, neutrophil infiltration occurred around the hematoma, and the expression of IL-1β, TNF-α and pro-inflammatory protease increases significantly [47]. These inflammatory factors and proteins can cause more inflammatory cells, such as microglia cell aggregation, which produces a more significant inflammatory response and directly leads to BMEC injury [48]. Here, significantly increased microglial/macrophage was accumulated in the brain lesions, and the pro-inflammatory factors such as IL-1β and TNF-α were also overexpressed in brain lesions of SAH rats. However, overexpressing circARF3 significantly abated microglial/macrophage activation and inflammation. Interestingly, previous studies have confirmed that circARF3 has anti-inflammatory effects [18, 19], while this study testified that circARF3 has regulatory effects in neuroinflammatory responses and can be a potential therapeutic target.

Disorders of miRNAs are associated with a variety of neurological diseases, including cerebral hemorrhage [49]. Similar to circRNAs, miRNAs also have significant regulatory effects on BBB integrity destruction and neuroinflammatory response secondary to SAH. For instance, serum miR-126-3p levels are down-regulated in patients with cerebral hemorrhage, and overexpressing miR-126-3p significantly reduces the BBB permeability in the bleeding area of intracerebral hemorrhage (ICH) rats [50]. Similarly, the miR-27a-3p mimic treatment impedes neuronal apoptosis and microglial activation in the hemorrhagic lesions of SAH rats and alleviates the BBB damage and neurological impairment [51]. In this study, we investigated the regulation of miR-31-5p on BMEC function and microglial reaction to OGD/R. It turned out that miR-31-5p aggravated OGD/R-mediated BMEC permeability and microglia-mediated inflammation, indicating that miR-31-5p had a pro-inflammatory effect. In fact, miR-31-5p has been shown in several studies to aggravate endothelial cell injury. For example, cigarette smoke extract (CSE) promotes cell apoptosis, inflammation, and oxidative stress in human pulmonary microvascular endothelial cells (HPMECs), and miR-31-5p aggravates this effect [52]. In this study, we testified that circARF3 sponged and dampened miR-31-5p expression as a competitive endogenous RNA (ceRNA). In terms of effect, circARF3 can significantly attenuate the destruction of BMEC integrity by targeting miR-31-5p and the promotion of microglial inflammation, which further deepens our understanding of the circARF3-mediated protective effect in SAH.

Toll-like receptor 4 (TLR4) is a pattern recognition receptor and contributes to inflammatory responses mediated by microglia, neurons, astrocytes, endothelial cells, etc. [53]. After being activated by its ligand, TLR4 can activate NF-κB through a MyD88-dependent pathway, which produces TNF-α and IL-1β [54]. In this study, we observed that SAH promoted the MyD88-NF-κB pathway activation, while overexpressing circARF3 attenuated the pathological changes both *in vivo* and *in vitro*. Conversely, the up-regulation of miR-31-5p activated MyD88-NF-κB, and this effect was rescued by circARF3 overexpression. Interestingly, previous studies have reported that miR-31-5p is up-regulated after NF-κB pathway activation and exacerbates the inflammatory response [26], while pro-inflammatory factors such as IL-6 or TNF-α contribute to the up-regulation of miR-31-5p [55]. These findings suggest that miR-31-5p may form a positive feedback loop with the MyD88-NF-κB pathway. Therefore, we hypothesized that circARF3 could improve the secondary brain injury after SAH by downregulating the miR-31-5p-MyD88-NF-κB pathway.

Overall, our study suggests that circARF3 sponges miR-31-5p to inactivate the MyD88-NF-κB pathway, maintain endothelial function, and reduce SAH-induced BBB destruction, neuronal apoptosis, and microglial inflammation. This study further reveals the potential mechanism of circARF3 in alleviating SAH-induced BBB injury, which provides a new target for the study of cerebral hemorrhage and stroke treatment. However, further studies are needed to demonstrate the value of the circARF3-miR-31-5p-MyD88-NF-κB axis in SAH treatment.

## Supplementary Material

Supplementary Figures
