# Supplementary material for: Up-regulation of circARF3 reduces blood-brain barrier damage in rat subarachnoid hemorrhage model via miR-31-5p/MyD88/NF-κB axis
Source: Aging (Albany NY). 2021 Sep 12;13(17):21345–63. doi: 10.18632/aging.203468 (PMC8457610; doi:10.18632/aging.203468)
Supplement: Supplementary Figures [file aging-13-203468-s001.pdf]

SUPPLEMENTARY FIGURES

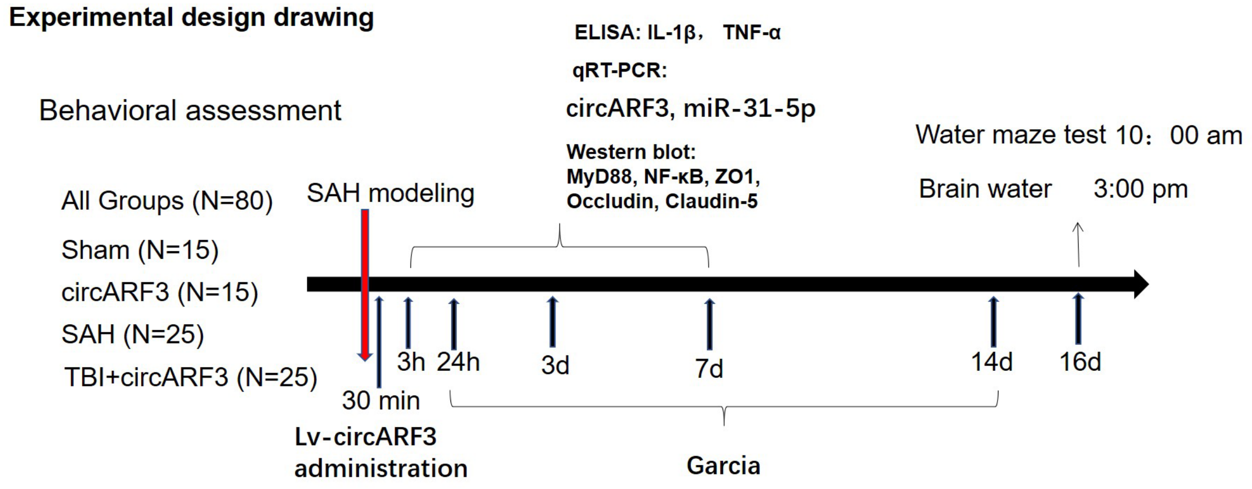

Supplementary Figure 1. The animal experiment procedures were shown.

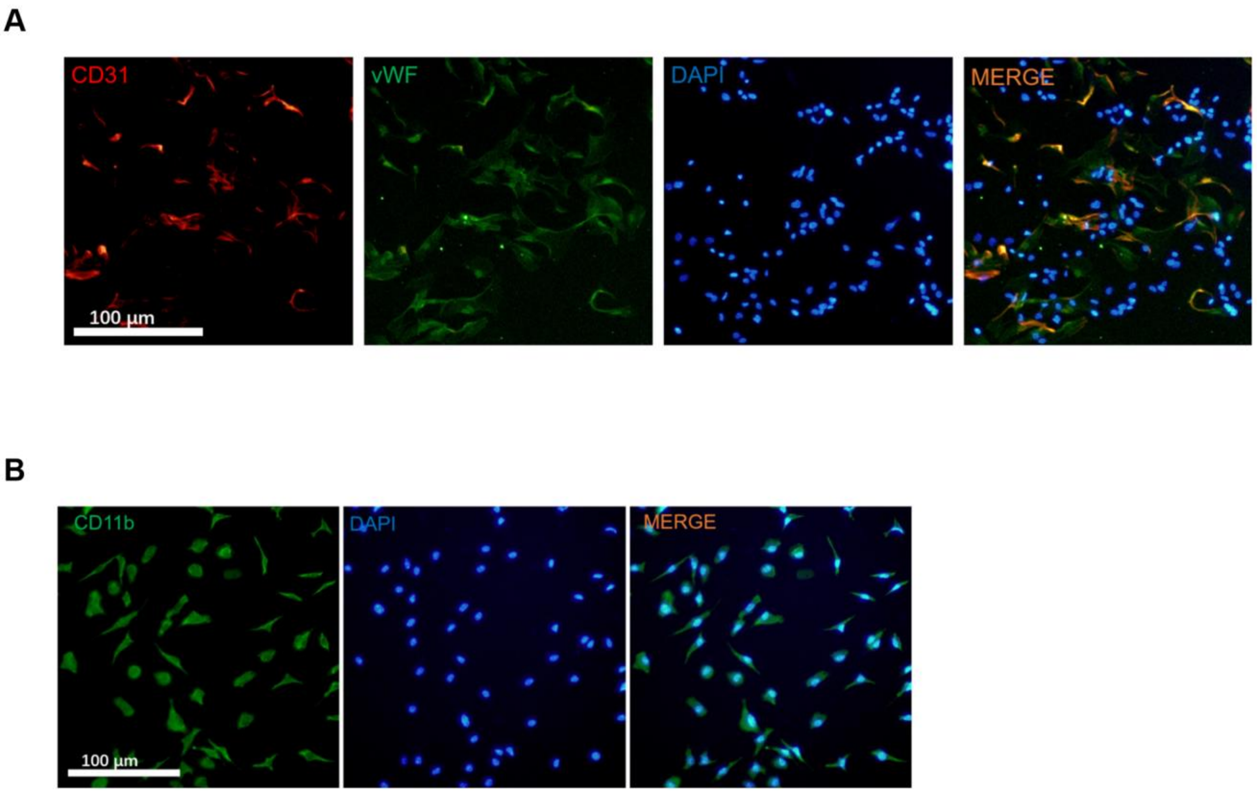

Supplementary Figure 2. (**A**) Cell immunofluorescence was used to identify primary BMECs labeled by CD31 (red) and vWF (green). (**B**) Cell immunofluorescence was used to identify microglia labeled by CD11b (green).
